# Advancing Mycotoxin Detection: Multivariate Rapid Analysis on Corn Using Surface Enhanced Raman Spectroscopy (SERS)

**DOI:** 10.3390/toxins15100610

**Published:** 2023-10-12

**Authors:** Allison Gabbitas, Gene Ahlborn, Kaitlyn Allen, Shintaro Pang

**Affiliations:** 1 Department of Microbiology and Molecular Biology, Brigham Young University, Provo, UT 84602, USA; agabbit2@byu.edu (A.G.); kallen45@byu.edu (K.A.); 2 Department of Nutrition, Dietetics and Food Science, Brigham Young University, Provo, UT 84602, USA; gene_ahlborn@byu.edu

**Keywords:** surface enhanced Raman spectroscopy, nanotechnology, label-free, mycotoxin, aflatoxin B1, zearalenone, ochratoxin A

## Abstract

Mycotoxin contamination on food and feed can have deleterious effect on human and animal health. Agricultural crops may contain one or more mycotoxin compounds; therefore, a good multiplex detection method is desirable to ensure food safety. In this study, we developed a rapid method using label-free surface-enhanced Raman spectroscopy (SERS) to simultaneously detect three common types of mycotoxins found on corn, namely aflatoxin B1 (AFB1), zearalenone (ZEN), and ochratoxin A (OTA). The intrinsic chemical fingerprint from each mycotoxin was characterized by their unique Raman spectra, enabling clear discrimination between them. The limit of detection (LOD) of AFB1, ZEN, and OTA on corn were 10 ppb (32 nM), 20 ppb (64 nM), and 100 ppb (248 nM), respectively. Multivariate statistical analysis was used to predict concentrations of AFB1, ZEN, and OTA up to 1.5 ppm (4.8 µM) based on the SERS spectra of known concentrations, resulting in a correlation coefficient of 0.74, 0.89, and 0.72, respectively. The sampling time was less than 30 min per sample. The application of label-free SERS and multivariate analysis is a promising method for rapid and simultaneous detection of mycotoxins in corn and may be extended to other types of mycotoxins and crops.

## 1. Introduction

Mycotoxins are toxic secondary metabolites produced by various types of fungi, such as Aspergillus, Penicillium, and Fusarium [1]. They thrive under stressed environmental conditions, such as high temperatures and humidity, and commonly contaminate agricultural crops [2]. Optimal mycotoxin production typically occurs in climates where temperatures are between 25 and 35 °C with higher levels of rainfall [3]. These toxins pose a significant threat to human and animal health when they contaminate food and feed [4]. Mycotoxins can cause a range of adverse health effects, including acute and chronic toxicity, carcinogenicity, immunosuppression, and even death [5]. The severity of these effects depends on factors such as the type of mycotoxin, level of exposure, and individual susceptibility. Over 400 compounds have been recognized as mycotoxins to date and ongoing toxicological studies and advancements in analytical tools continue to expand this list [6,7,8].

Aflatoxin, ochratoxin, and zearalenone are some of the more prominent groups of mycotoxins related to public health and agroeconomics [9]. These mycotoxins can be found in food and feed products, including cereals (such as corn, wheat, barley, and oats), nuts, spices, dried fruits, and animal feed [10]. Specifically, aflatoxin B1 (AFB1) is a potent mycotoxin and has been classified as a Group 1 carcinogen [11]. It is produced mainly by *Aspergillus flavus* and *Aspergillus parasiticus* [12]. Ochratoxin A (OTA), which is produced by *Aspergillus* and *Penicillium* species, is classified as Class 2B, a class of probable human carcinogens, by the International Agency for Research on Cancer (IARC) [13]. Zearalenone (ZEN), which has a chemical structure similar to the female sex hormone estrogen, is produced by various *Fusarium* fungi species [14] and has been linked to various adverse health effects in humans, including precocious puberty and breast cancer [15,16].

The detection and quantification of mycotoxins in food and feed samples are critical for ensuring food safety and preventing outbreaks of mycotoxicosis, so establishing a reliable and efficient method is also essential for regulatory compliance. Traditionally, the analytical methods employed usually require preliminary steps prior to analysis, including toxin extraction from the matrix, a clean-up procedure to remove interfering elements prior to detection, and quantification using appropriate analytical instrumentation [17]. To date, the most common instrumentation used has included chromatographic systems coupled with highly sensitive detection systems such as liquid chromatography (LC) or high-pressure liquid chromatography (HPLC) coupled with a signal transduction mechanism such as fluorescence, ultraviolet, or mass spectrometry [18,19,20,21]. However, these conventional methods for mycotoxin analysis are time-consuming, expensive, and often require extensive sample preparation.

Rapid method alternatives have also been explored and employed, including immunological-based methods such as enzyme-linked immunosorbent assay (ELISA) [22,23] and lateral flow detection (LFD) [24]. While these methods are simpler and more rapid, there are some drawbacks, including the possibility of cross-reactivity and dependence on matrix type for sensitivity and accuracy [25,26]. Furthermore, these methods may have less sensitive detection limits and often rely on reporter molecules that are either attached to or react with the target analyte and can result in false positives [27]. Another challenge is the constraint in multiplex detection capabilities due to specificity issues, including using multiple antibodies in one single test [28,29]. As it is plausible for food and feed to be contaminated by multiple mycotoxins simultaneously, it is advantageous to develop sensitive and rapid methods that will accurately detect multiple mycotoxins at the same time.

In recent years, surface-enhanced Raman scattering (SERS) has emerged as a powerful analytical technique for the rapid and sensitive detection of various food contaminants, including pesticides [29,30], allergens [31,32], microplastics [33], and even pathogenic bacteria [34]. SERS utilizes the combination of Raman spectroscopy and nanotechnology to provide unique chemical and biochemical fingerprints for low levels of target analytes. Raman spectroscopy studies the molecular vibrations caused by inelastic light scattering, which typically provides a weak signal. However, placement of a sample near (~10 nm) or on roughened and noble metal nanosubstrates can enhance the Raman signals tremendously due to the large electromagnetic field induced by the excitation of the localized surface plasmon resonance (LSPR) [35]. This allows users to detect analytes at very low concentrations.

**Figure 1:**
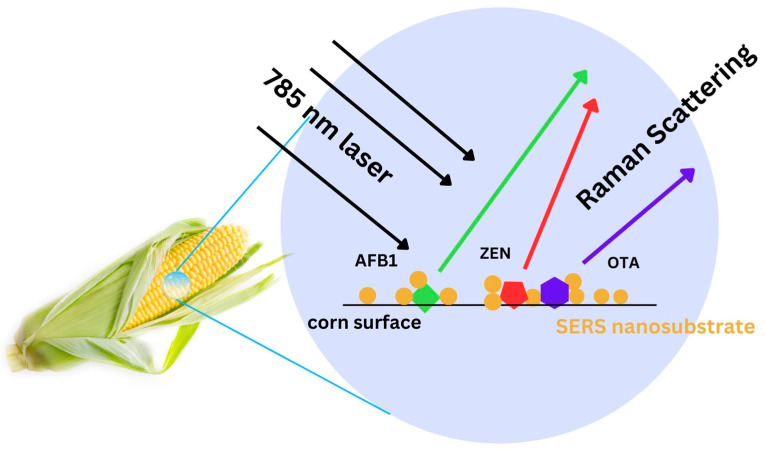
Schematic illustration of the developed method used to detect mycotoxin analytes (i.e., AFB1, ZEN, and OTA) on corn; SERS nanosubstrate was applied on the agricultural crop (i.e., corn) and mixed briefly with mycotoxins present on the surface. Raman spectral data were acquired using a Raman microscope equipped with a 785 nm laser.

SERS has been explored as a detection tool for mycotoxins in recent years [17,36]. However, many of these studies employed complicated SERS-active substrate fabrication that require highly specialized skills and sophisticated equipment, thus making them less accessible for wider audiences and application [37,38]. Additionally, those that focused on more widely available SERS substrates were either focused on individual mycotoxin type detection [39] or relied on secondary labeling with a Raman reporter molecule [40]. In this study, we present a simple, rapid, and yet novel label-free SERS method for the simultaneous detection and identification of multiple mycotoxins (i.e., AFB1, ZEN, and OTA) on corn. Our approach utilizes simple and colloidal gold nanoparticles as SERS nanosubstrates (Figure 1), a reference spectra library of the targets of concern, and multivariate statistical analysis to extract useful information from sample SERS spectra, thus enabling accurate identification of the mycotoxins present in corn samples. As far as we know, this is the first study that reports the simultaneous detection of multiple mycotoxins on corn using label-free SERS with colloidal gold nanoparticles.

## 2. Results and Discussion

The results of this study are categorized into three sub-sections: first, the identification and characterization of AFB1, ZEN, and OTA; second, the limit of detection of the method used to analyze each mycotoxin; and third, the simultaneous detection of mycotoxins.

### 2.1. Identification and Characterization of AFB1, ZEN, and OTA Using Label-Free SERS

**Figure 2:**
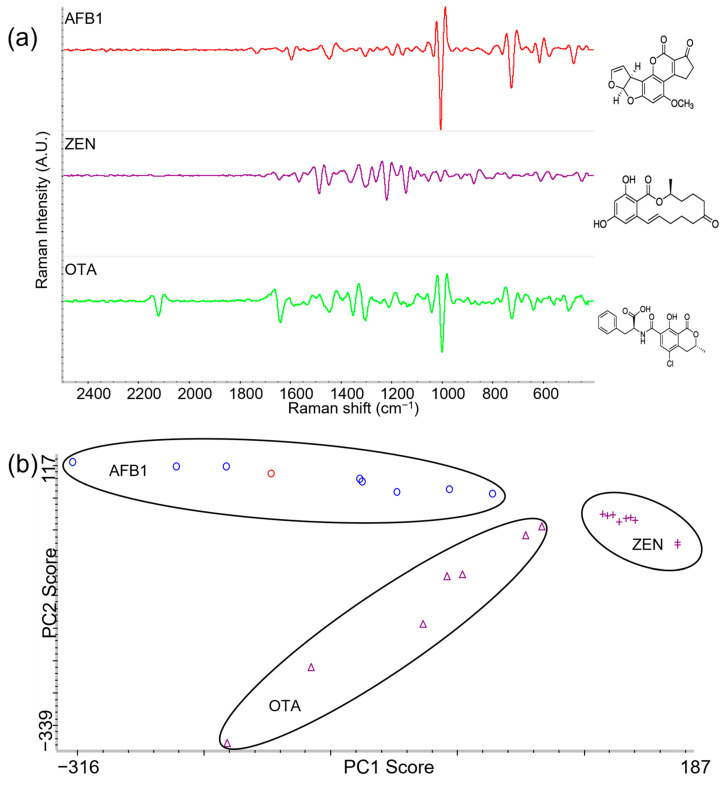
SERS identification and discrimination of mycotoxins used in this study: (**a**) second derivative Raman spectra of aflatoxin B1 (AFB1), zearalenone (ZEN), and ochratoxin A (OTA) and their respective chemical structure and (**b**) principal component analysis comparing the second derivative Raman spectra of the three mycotoxins (AFB1, ZEN, and OTA) against each other.

To create a reference for the identification of AFB1, ZEN, and OTA in this study, stock solutions of AFB1, ZEN, and OTA were mixed with SERS nanosubstrate and subsequently placed on corn for data acquisition. Figure 2a displays the second derivative SERS spectra and the chemical structure corresponding to each mycotoxin (raw spectra are shown in Figure S1). Second derivative transformation was applied to remove baseline deviations and separate overlapped peaks, which enhances the spectral resolution and interpretation of results [41,42]. It is clearly demonstrated in the figure that each mycotoxin has its own unique Raman fingerprints where each peak correlates with a specific functional group or vibration [43]. To further highlight statistical differences between SERS spectra of the three mycotoxins, multiple spectral data points were acquired for each mycotoxin and principal component analysis (PCA) was performed (Figure 2b). By leveraging PCA, Raman shifts were decomposed through linear combinations to produce a reduced number of variables expressed through vibrational information [44]. Figure 2b shows distinct separation and grouping of the three mycotoxin spectra after PCA conversion. ZEN exhibited the tightest grouping but AFB1 and OTA grouping was still clearly distinct. Our results correlate with other findings where SERS spectra conversion to PCA was successfully used for the rapid identification of mycotoxins, including aflatoxins, deoxynivalenol, fumonisin B1 [45,46], and bacteria [47,48].

Table 1 provides a summary of the prominent Raman shifts and corresponding vibrational mode peak assignments of AFB1, ZEN, and OTA, as displayed in Figure 2a. For AFB1, eight unique peaks were identified. Of note, the 615 cm^−1^ peak was assigned to the ring deformation of the compound and 1272 cm^−1^ was associated with the β(C–H) ring deformation. The 1595 cm^−1^ peak was attributed to *ν*(C–C) and *ν*(C–C–C), which corresponds with other Raman studies on AFB1 [49,50]. Five unique Raman peaks for ZEN were each assigned to a specific functional group/vibration, i.e., 876 cm^−1^, 1142 cm^−1^, 1259 cm^−1^, 1484 cm^−1^, and 1563 cm^−1^. A prominent peak, 1142 cm^−1^, was associated with the β(C–H)(ring), β(C–H)(–CH_3_), while the peak at 1484 cm^−1^ was assigned to *ν*(C_7_=C_8_), ring deformation. For OTA, the 1040 cm^−1^ peak represented C-Cl stretching while the 1350 cm^−1^ peak was assigned to δCH_3_. Among the four Raman peaks identified for OTA, it is worth noting that 723 cm^−1^ and 1000 cm^−1^ were exhibited in AFB1 as well, where 723 cm^−1^ corresponded to the C–H out-of-plane bending and 1000 cm^−1^ corresponded to the β(C–O) and *ν*(C–C) mode. The observation of having two compounds with similar peaks is not uncommon as different compounds may have similar functional groups and chemical bonds [51]. However, the combination of all the Raman peaks within the evaluated Raman shifts result in a unique fingerprint combination that can be differentiated using chemometric tools. These data show that the characterization of these target compounds using label-free SERS gives greater understanding of their molecular structure, which also provides a way to detect them.

### 2.2. Limit of Detection (LOD) of Mycotoxins: AFB1, ZEN, and OTA

**Figure 3:**
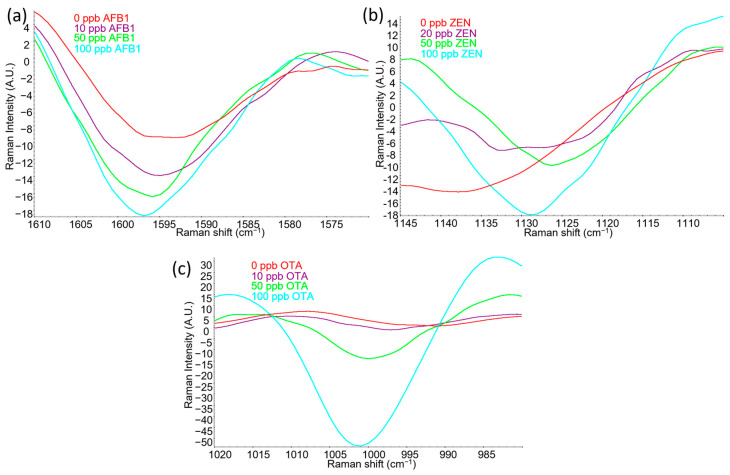
Second derivative Raman spectra of the target capture peak for the respective mycotoxin compounds at varying concentration levels: (**a**) Aflatoxin B1 (AFB1) between 1570 and 1610 cm^−1^ at 0, 10, 50, and 100 ppb (0, 32, 160, and 320 nM); (**b**) Zearalenone (ZEN) between 1105 and 1145 cm^−1^ at 0, 20, 50, and 100 ppb (0, 31, 157, and 314 nM); (**c**) Ochratoxins A (OTA) between 980 and 1020 cm^−1^ at 0, 10, 50, and 100 ppb (0, 25, 124, and 248 nM).

Samples with varying mycotoxin concentrations from 10–100 ppb (32–320 nM) were evaluated and are shown in Figure 3. Increasing concentrations of the mycotoxins of interest, AFB1, ZEN, and OTA, resulted in increased intensities at certain Raman shifts, specifically around 1597 cm^−1^ (Figure 3a), 1130 cm^−1^ (Figure 3b), and 1002 cm^−1^ (Figure 3c), respectively. These increased intensity peaks corresponded with Raman shifts that were previously identified and characterized in Table 1. The relationship between SERS peak intensity and actual target analyte concentration has been well documented and has been shown to allow the quantification of trace chemicals [47,48]. 

In addition to the increased peak intensity, slight peak shifts were observed as well. The negative control, i.e., samples without mycotoxins, had flat or rounder peaks around 1593–1597 cm^−1^, 1135–1140 cm^−1^, and 997–1002 cm^−1^ but as the mycotoxin concentration on corn became increasingly higher, the shape of the peaks became sharper and more distinct. In some studies, the shifts in the Raman peaks were explained to be the result of a change in the adsorption or orientation of the molecules on the SERS active nanosubstrate [49]. While this may have contributed to the overall peak shift, the background signals of the corn could also have contributed to the results as we can see the gradual shift in the SERS peak as the mycotoxin concentration increased. In particular, ZEN had the most significant shift in peak among the three mycotoxins tested (Figure 2b). There was not a clear peak in the negative control between 1135 and 1140 cm^−1^, but a pattern of more distinct and intensified peak was observed as mycotoxin concentration increased. AFB1 could also be identified clearly from the negative control due to a stronger intensity and slightly shifted peak around 1597 cm^−1^ when AFB1 concentration increased. OTA was more straightforward as the negative control had little, if any, observation of a peak within the OTA induced peak around 1002 cm^−1^.

**Figure 4:**
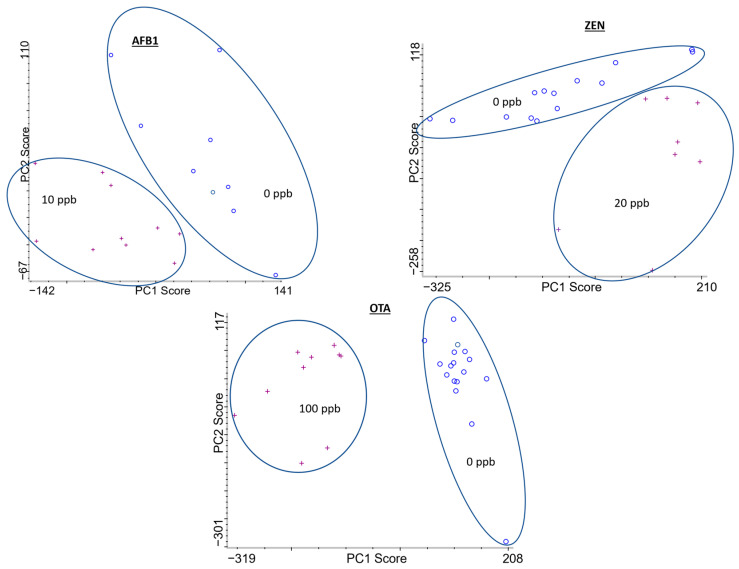
Principal component analysis comparing the second derivative Raman spectra of a negative control (0 ppb) against AFB1, ZEN, and OTA at 10 ppb, 20 ppb, and 100 ppb, respectively. Clear separation between data clusters of each variable is observed, indicating a notable difference.

To assess the limit of detection (LOD) of the mycotoxins on corn samples, principal component analysis (PCA) was applied. This discriminant analysis enabled the evaluation of SERS spectra for variance within a class and between classes. The LOD was determined to be the lowest concentration of the data cluster that could be separated from the negative control [55]. As shown in Figure 4, the LOD for AFB1, ZEN and OTA were 10 ppb (32 nM), 20 ppb (63 nM), and 100 ppb (248 nM), respectively, as there was clear separation of the data clusters between the control, i.e., no mycotoxin on corn and the reported mycotoxin concentration. While the maximum level allowed in corn and other food and feed is dependent on a variety of factors such as the regulatory entities of that region and the specific type of mycotoxins, the sensitivity of this method is comparable to other promising analytical methods [17]. While there are other sensitive analytical methods that have reported better limits of detection [52,56], these methods either require sophisticated equipment to operate or require specialized skills to fabricate chemically synthesized nanomaterials that are not widely available. Future studies can be focused on improving sensitivity even further through strategies such as optimizing the extraction procedure and the interaction with SERS substrate. The method used in this study, however, uses a simple approach and utilizes more widely available materials to accomplish high sensitivity. Furthermore, the label-free approach eliminates a labeling step which preserves sample integrity and allows the ability for simultaneous detection without the need for multiple labels [57], which is being presented in more detail in the next section.

### 2.3. Simultaneous Detection of Mycotoxins on Corn

**Figure 5:**
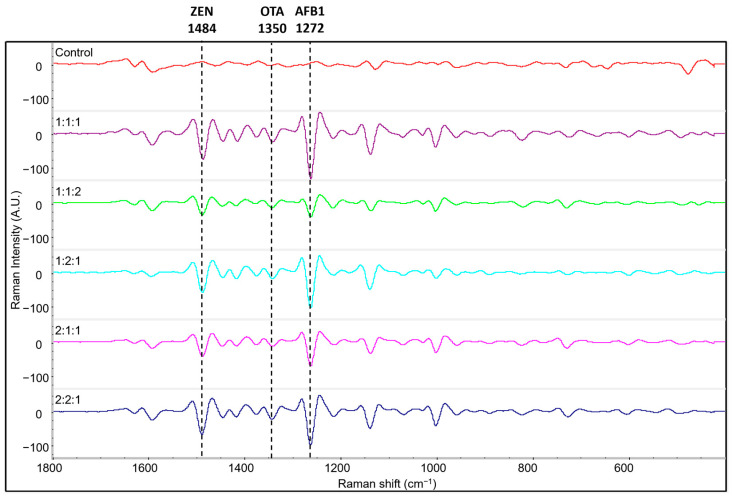
Second derivative Raman spectra of three mycotoxins spiked on corn at different concentration ratios in the following order AFB1:ZEN:OTA. Actual concentrations of each mycotoxin are shown in Table S1 (refer to Section 4 for more details). The dashed lines on the figure highlight identifying peaks of each mycotoxin present in each sample.

To demonstrate the feasibility of simultaneous detection of mycotoxins using the developed label-free SERS method, AFB1, ZEN, and OTA, were spiked onto corn at varying concentration ratios. Their respective SERS spectra were then collected and analyzed. Figure 5 shows the SERS spectra of corn with spiked concentration ratios reported in the following order: AFB1:ZEN:OTA. Table S1 provides detail of all the mycotoxins and concentrations tested. For example, 1:1:1 was equivalent to 1 ppm:1 ppm: 1 ppm, 1:1:2 was equivalent to 0.75 ppm:0.75 ppm:1.5 ppm, etc. These samples produced distinct peaks on all samples compared to the control which was corn containing no mycotoxins. Based on the initial identification and characterization study of these mycotoxins, these spectra contained several identifiable peaks corresponding to AFB1, ZEN, and OTA. Among these are known peaks at 1272 cm^−1^ for AFB1, 1484 cm^−1^ for ZEN, and 1350 cm^−1^ for OTA, as explained in Section 2.1. Similar analytical procedures and results have been achieved using label-free SERS to perform simultaneous detection. For example, Sun et al. 2019 was able to utilize a SERS-microfluidic droplet platform to simultaneously detect multiple metabolites, such as lactate, ATP, and pyruvate, at the single cell level using characteristic Raman peaks representing these metabolites [58]. Hassan et al. 2022 simultaneously detected two fungicides, carbendazim (CBZ) and thiabendazole (TBZ), using the 1224 cm^−1^ and 778 cm^−1^ peaks that correlated with their specific target analyte presence [59]. Our results on corn also show a similar pattern and indicate that simultaneous detection of AFB1, ZEN, and OTA can be achieved on food and feed.

The quantification capability of this method was also evaluated to determine whether it is possible to predict the concentrations of multiple mycotoxins in corn. Figure S2 shows the overlaid spectra of the same SERS spectra data displayed in Figure 5 to emphasize the peak intensity differences resulting from the different concentrations of mycotoxins added to each variable. The SERS peak intensities of target analytes are directly correlated with the analyte concentrations [60] and thus can be used for quantification studies. In many instances, spectroscopic studies leverage chemometric tools to determine correlations between peak intensities and known concentrations to establish a standard curve [61,62,63]. In this study, partial least squares regression, a multivariate statistical analysis tool, was used to develop the model to predict target mycotoxin concentrations. 

**Figure 6:**
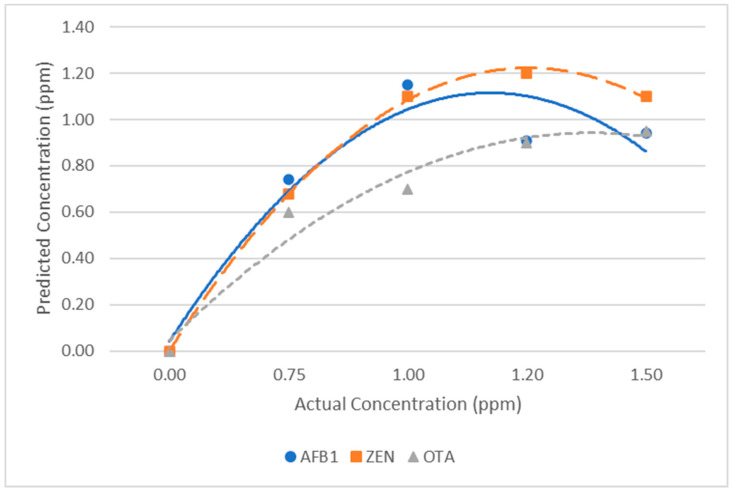
Scatterplot of predicted concentration versus actual concentration of AFB1, ZEN, and OTA on corn using label-free SERS and partial least squares (PLS) regression analysis.

Figure 6 presents a summary of the relationship between the predicted concentration and actual concentration of AFB1, ZEN, and OTA on corn using label-free SERS and partial least squares (PLS) regression analysis. While there is noticeably a positive trend of higher predicted concentration when the actual concentration is higher, it is worth noting that the predicted concentration reached its peak at 1.0 ppm (3.2 µM) for AFB1 and 1.2 ppm (3.77 µM) for ZEN. The strength of the linear relationship between the predicted concentration and actual concentration was also slightly different for AFB1, ZEN, and OTA, with a correlation coefficient of 0.74, 0.89, and 0.72, respectively (Figure S3). Although these values indicate there is a strong association, it is important to acknowledge that there are other developed analytical techniques that can have correlation coefficients much closer to one [20,38]. 

A potential reason as to why the method tested did not yield a higher correlation coefficient could be due to the non-homogeneity and porosity of the corn surface where the mycotoxin solution was deposited, resulting in variability in the actual mycotoxin amount extracted. It could also have been due to the non-uniform distribution of SERS nanosubstrate on the sample, which could have impacted the enhancement factor of the Raman signal amplification [64]. To improve the quantification capability for future studies, more attention should be given to investigating and improving the extraction procedure, such as the extraction time and volume. SERS nanosubstrates that provide a more uniform distribution could also be used. For example, Qu et al., 2017, developed a simple SERS substrate called silver nanoparticle (AgNP) mirror substrate that yielded a very high coefficient of determination (0.9981) compared to AgNP aggregates [65] and has been successfully used for application on food surfaces [66,67]. The results of this study suggest the quantification capability needs to be improved and thus the method may be more of a rapid screening and qualitative measure. Nevertheless, the focus of this study was to develop and evaluate a simple and rapid label-free SERS method using unsophisticated and well-established materials, such as colloidal gold nanoparticles that can be easily purchased to determine its feasibility for real applications. As far as we know, this is the first study that reports the simultaneous detection of multiple mycotoxins on corn using label-free SERS with colloidal gold nanoparticles.

## 3. Conclusions

In summary, a simple and novel label-free SERS method was developed for the simultaneous detection of three mycotoxins, namely AFB1, ZEN, and OTA, on corn. These mycotoxins exhibited unique chemical fingerprints allowing for the identification, characterization, and differentiation of the target analytes. The limit of detection was determined to be between 10 and 100 ppb (32–248 nM) using principal component analysis, demonstrating the ability to detect trace levels of AFB1, ZEN, and OTA, which is comparable to the sensitivity of other promising analytical methods [17]. While lower LODs have been reported with some other methods [52,56], these methods require sophisticated equipment or specialized skills. The method used in this study, however, uses a simple approach and utilizes more widely available materials to accomplish high sensitivity. The simultaneous detection of all three mycotoxins was also made possible without the need for secondary markers as the distinct Raman peaks associated with the intrinsic chemical fingerprints of AFB1, ZEN, and OTA were leveraged. Further investigation should be conducted to optimize the reliability of the method for quantitative determination. Nevertheless, this study demonstrates the promising application of this method for rapid and simultaneous detection of multiple mycotoxins in corn, which can potentially be extended to detecting other types of mycotoxins in food and feed.

## 4. Materials and Methods

### 4.1. Materials

All chemicals were of analytical reagent grade and were purchased through Fisher Scientific (Waltham, MA, USA) unless otherwise noted. Pure reference standards of the three studied mycotoxins: aflatoxin B1 (AFB1), zearalenone (ZEN), and ochratoxin A (OTA) were purchased through Sigma-Aldrich (St. Louis, MO, USA) with an original concentration of 20 µg/mL, 50 µg/mL, and 50 µg/mL, respectively. Gold nanoparticles (colloidal, citrate-capped AuNPs, 50 nm at original concentration of 0.1 mg/mL) were purchased from a commercially available source (Thermo Scientific, Waltham, MA, USA).

### 4.2. Sample Preparation for SERS Characterization of Mycotoxins 

To prepare samples containing each mycotoxin for SERS identification and characterization, a simple protocol developed by Hou et al. 2015 was leveraged [68]. Briefly, 2 µL of AuNPs and 2 µL of each reference mycotoxin stock solution was deposited onto parafilm and mixed briefly by pipetting for ~20 s. Then, 2 µL of the mixed solution was extracted and placed on a gold-plated (1″ × 3″, 50 nm gold film) microscope slide (Platypus Technologies, Fitchburg, WI, USA). Several solvents, including water, methanol, and acetonitrile, were tested to determine the best way to apply and extract these mycotoxins. Methanol was found to be the best solvent and was used in subsequent experiments. Deposited droplets were then allowed to dry before SERS analysis, forming a ring of aggregated AuNPs bound to the mycotoxin.

### 4.3. Detection of Mycotoxins on Corn

Mycotoxin working solutions were prepared to the following concentrations: 10 ppb, 20 ppb, 50 ppb, and 100 ppb (32, 64, 160, and 320 nM) by dilution of the reference standard with methanol. The pure solvent was used as a negative control. Corn samples were provided by H Abbas from USDA (United States Department of Agriculture) and were verified prior to the study to be free from AFB1, ZEN, and OTA. To simulate known levels of mycotoxins on corn kernels, samples were spiked with 2 µL of the mycotoxins solution at varying concentrations and allowed to dry. Then, 2 µL of AuNP solution was dropped on the corn surface and pipetted for ~20 s to mix in the AuNPs with mycotoxin previously spiked onto corn. Droplets were allowed to dry. A ring of aggregated AuNPs was deposited as the sample dried in preparation for SERS analysis. 

### 4.4. Simultaneous Detection of Multiple Mycotoxins

Five working solutions containing AFB1, ZEN, and OTA were prepared with varying concentrations of each mycotoxin. Table S1 shows the concentration of each mycotoxin present in the five working solutions, excluding the negative control. These working solutions were made by first preparing individual mycotoxin solutions at a concentration of 3 ppm (9.6 µM) and then by combining them in volume ratios of 1:1:1, 1:1:2, 1:2:1, 2:1:1, and 2:2:1. Corn kernels were spiked with 2 µL of each working solution and allowed to dry until no longer visibly wet. In total, 2 µL of AuNPs were then dropped on the corn surface and repetitively pipetted for ~20 s to mix in the AuNPs with the mycotoxins spiked onto corn; then, they were allowed to dry before SERS analysis. 

### 4.5. SERS Instrument and Data Analysis

The samples were analyzed using a DXR3 Raman spectro-microscope (Thermo Scientific, Madison, WI, USA). The following conditions were used for this study: a 10× confocal microscope objective (3 μm spot diameter and 5 cm^−1^ spectral resolution), 780 nm excitation wavelength, 10 mW laser power, and 50 μm slit width with a 1 s integration time. OMNIC™ software version 9.13 was used to operate the Raman instrument and to obtain Raman spectra. Then, 15–25 spots were selected randomly on each sample for analysis by the Raman instrument within the areas having visible dark spots indicating AuNP aggregation. SERS spectra were then obtained within the range of 550 to 2000 cm^−1^. A range of peak intensities was captured. Generally, spectra below 1000 (AU) were considered very weak signals and did not have any AuNP or mycotoxins peaks associated with them, suggesting that no SERS nanosubstrate enhancements were captured. For a more refined and purposeful analysis, a rule was made to remove these weak spectra without exception. The spectrum above 1000 (AU) collected from each point was then averaged. SERS spectral data were analyzed using TQ Analyst software (version 9.13, Thermo Scientific, Madison, WI, USA). Second derivative transformation and smoothing were applied to reduce spectral noise and to separate overlapping bands. The variances of spectral data between spots and samples were then assessed using principal component analysis (PCA). This method focuses on a multidimensional data set to the most dominant features while removing random variation so that the principal components can be used to capture the variation between spectra. This discriminant analysis is thus useful in evaluating SERS spectra for variance within a class and between classes. If two data clusters (classes) did not overlap, then it meant they were significantly different at the *p* = 0.05 level [42,69]. The limit of detection, LOD, was determined to be the lowest concentration of the data cluster that could be separated from the negative control in the PCA plot. Partial least squares (PLS), a multivariate analysis model, was also applied to evaluate the linear relationship between the predicted concentration and actual concentration. This model was self-validated using the leave-one-out validation method where all but one sample was used to build the calibration model and repeats for each sample in the data set. The correlation coefficient, root mean square error of calibration (RMSEC), and root mean square error of prediction (RMSEP) were also calculated to evaluate the quality of the model.

## Figures and Tables

**Table 1:** Summary of Raman shifts and corresponding vibrational mode peak assignments of AFB1, ZEN, and OTA.

AFB1	ZEN	OTA	Peak Assignments	Reference
1595			*ν*(C–C) and *ν*(C–C–C)	[49]
1561	1563		*ν*(C–C) and ring deformation	[49]
	1484		*ν*(C_7_=C_8_), ring deformation	[52,53]
		1350	δCH_3_	[36,54]
1305			A(C–H_2_)(ring)(C–H)	[49]
1272			β(C–H) ring deformation	[49]
	1259		β(C–H_2_) (ring)	[52,53]
	1142		β(C–H)(ring), β(C–H)(–CH_3_)	[52,53]
		1040	C-Cl stretching	[36,54]
1000		1000	β(C–O), *ν*(C–C)	[36,54]
927			Ring breath, *ν*(C–O)	[49]
	876		CH_2_ rocking	[52,53]
726		723	C–H out-of-plane bending	[36,54]
615			Ring deformation	[49]

## Data Availability

Further data are available in the Supplementary Information section.

## Supplementary Materials

The following supporting information can be downloaded at https://www.mdpi.com/article/10.3390/toxins15100610/s1. Figure S1: Raw SERS spectra of aflatoxin B1 (AFB1), zearalenone (ZEN), and ochratoxin A (OTA); Table S1: Summary of mycotoxin concentration cocktail that was applied onto corn; Figure S2: Overlaid second derivative Raman spectra of three mycotoxins spiked on corn at different concentration ratios in the following order: AFB1:ZEN:OTA; Figure S3: Partial least squares regression curve of AFB1, ZEN, and OTA using SERS spectra obtained from corn spiked with the three mycotoxins.
