# Supplementary material for: Advancing Mycotoxin Detection: Multivariate Rapid Analysis on Corn Using Surface Enhanced Raman Spectroscopy (SERS)
Source: Toxins (Basel). 2023 Oct 12;15(10):610. doi: 10.3390/toxins15100610 (PMC10610586; doi:10.3390/toxins15100610)
Supplement: Supplementary file 1 [file toxins-15-00610-s001.zip › toxins-2637551-supplementary.pdf]

Supplementary Information

The supplementary materials presented here provide details of some of the additional data that was collected to enhance the development of the analytical method developed to detect mycotoxins.

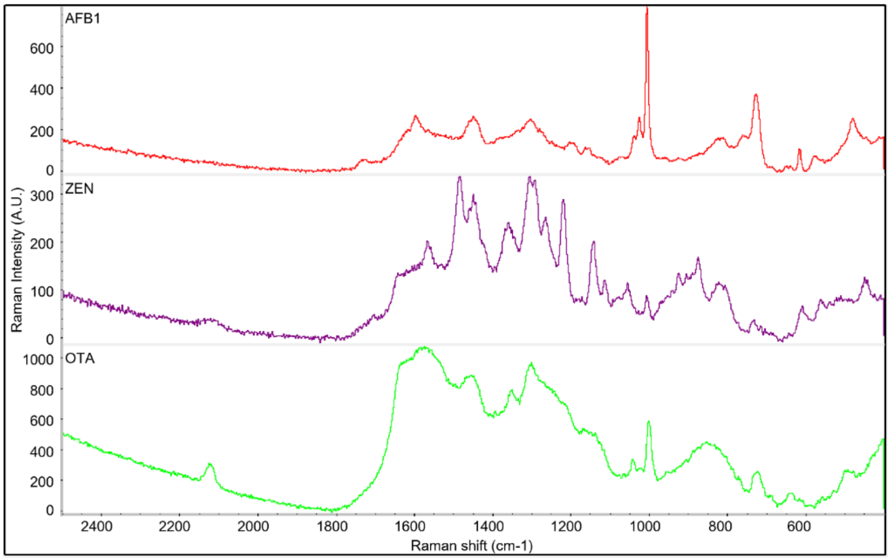

Figure S1. Raw SERS spectra of aflatoxin B1 (AFB1), zearalenone (ZEN), and ochratoxin A (OTA).

Table S1. Summary of mycotoxin concentration cocktail that was applied onto corn.

	**Mycotoxin Concentration (ppm)**		
**Variable**	**AFB1**	**ZEN**	**OTA**
T1	0.00	0.00	0.00
T2	1.00	1.00	1.00
T3	0.75	0.75	1.50
T4	0.75	1.50	0.75
T5	1.50	0.75	0.75
T6	1.20	1.20	0.60

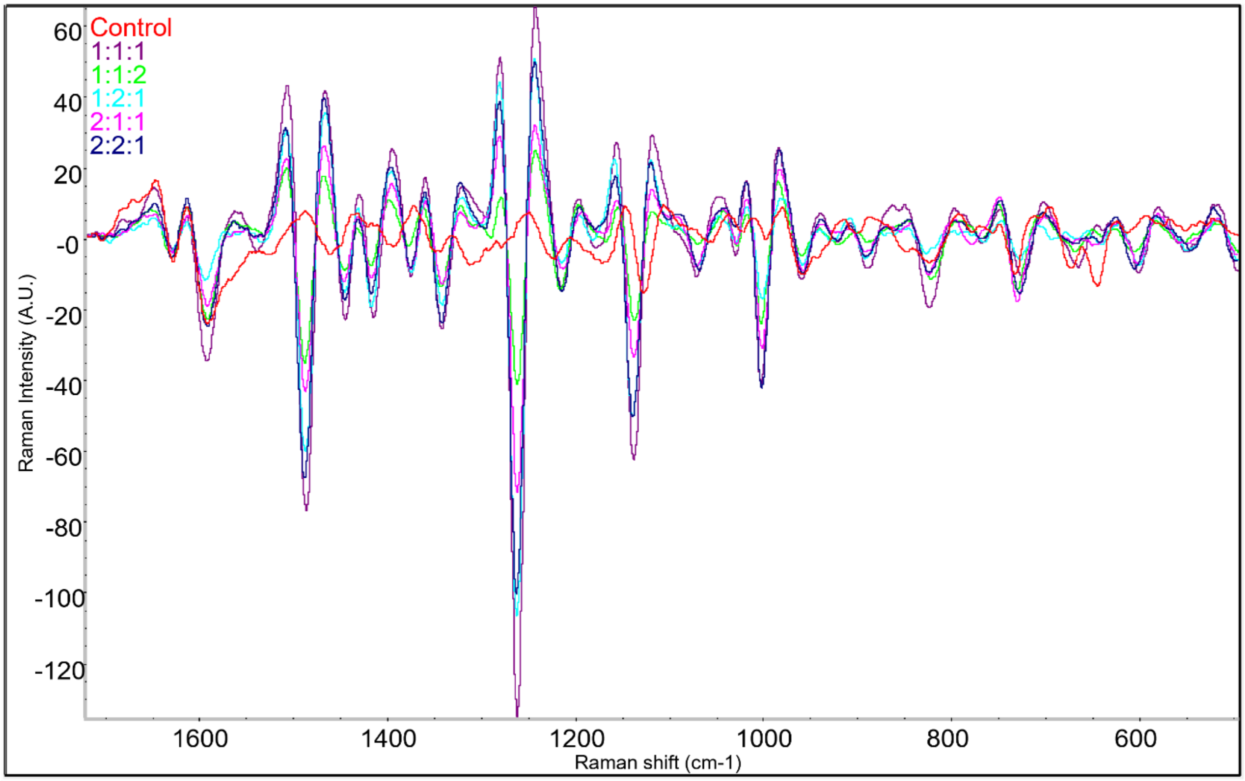

Figure S2. Overlaid second derivative Raman spectra of three mycotoxins spiked on corn at different concentration ratios in the following order – AFB1:ZEN:OTA.

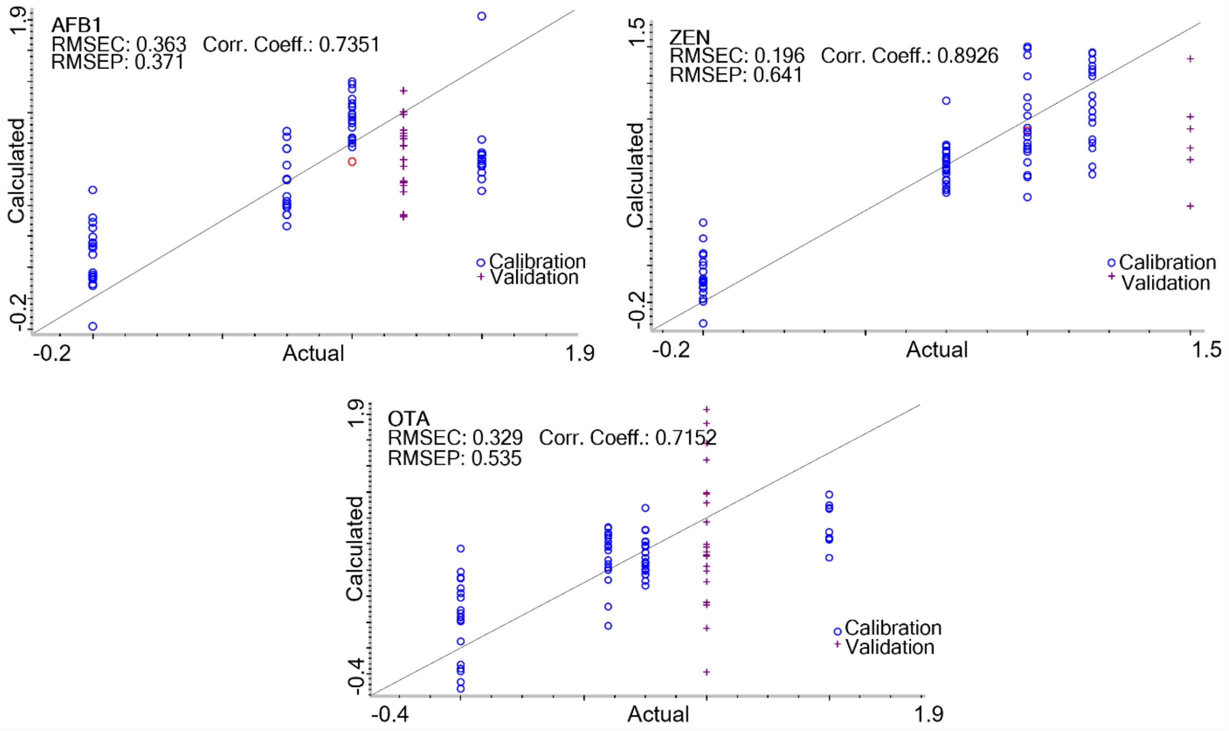

Figure S3. Partial Least Squares Regression curve of AFB1, ZEN and OTA using SERS spectra obtained from corn spiked with the three mycotoxins.
